# Big and Mini: A Promising Program to Link Generations to Cope With Social Isolation

**DOI:** 10.1093/geroni/igab046.2195

**Published:** 2021-12-17

**Authors:** Zhirui Chen, Ling Xu, Noelle Fields, Allen Zhou, Anthony Zhou, Aditi Merchant

**Affiliations:** 1 University of Texas at Arlington, Arlington, Texas, United States; 2 The University of Texas at Austin, Austin, Texas, United States; 3 The University of North Texas, Denton, Texas, United States

## Abstract

Introduction: Social isolation disproportionately affected older adults prior to and especially during the COVID-19 pandemic. To help older adults cope with social isolation, a new program “Big and Mini” was created in April 2020 to link young and older adults together (matched through a custom website developed for this program) and to help increase social connectivity through weekly phone calls. Using a survey with both closed and open questions, this study evaluated participant feedback three months after the program was launched. Methods: 63 Bigs (age 50+) and 53 Minis (age 18+) completed the survey. Stress compared to before COVID-19, social isolation, life satisfaction, intergenerational solidarity, and satisfaction with the program were measured for both the Big and Mini participants. Descriptive, bivariate correlation, group comparison and conventional content analyses were conducted. Results: Results showed that 38.1% of Bigs and 37.7% of Minis felt higher levels of stress than before COVID-19. Both Bigs and Minis had medium levels of social isolation. They also reported high levels of satisfaction with life, satisfaction with the program, and intergenerational solidarity. Content analysis suggested that the reasons to join or expectations of the program were curiosity, friendship, mutually beneficial intergenerational connections, and coping with loneliness. Both Bigs and Minis reported benefits from the learning and sharing opportunities that the program offered. Conclusions: The Big and Mini program offers a promising approach with mutual benefits for both Bigs and Minis. Strategies to improve the program and implications for other phone-based intergenerational programs are presented.

